# Is informal caregiving at odds with optimal health behaviour? A cross-sectional analysis in the caregiving partners of persons with spinal cord injury

**DOI:** 10.1080/21642850.2020.1838282

**Published:** 2020-11-02

**Authors:** Hannah Tough, Martin W.G. Brinkhof, Christine Fekete

**Affiliations:** a Swiss Paraplegic Research, Nottwil, Switzerland; b Department of Health Sciences and Medicine, University of Lucerne, Lucerne, Switzerland

**Keywords:** Caregivers, health behaviours, spinal cord injury, informal care, caregiver burden

## Abstract

**Background:**

The intricate relationship between caregiving and health may to some extent be determined by the burden and restrictions imposed on informal caregivers, and the impact these experiences have on health behaviours. It is assumed that a positive caregiver experience leads to health promoting behaviours in caregivers, whereas a negative experience induces the opposite. The objective of this study is to test these assumptions and to investigate the association between the caregiver experience and health behaviours in the caregiving partners of persons with severe physical impairment, due to spinal cord injury.

**Methods:**

Cross-sectional survey data from 133 couples of caregiving partners and persons with spinal cord injury living in Switzerland was used. We employed multivariable regression to evaluate the associations of the caregiver experience (objective and subjective caregiver burden, and satisfaction with caregiving) with health behaviours (physical activity, fruit and vegetable consumption, alcohol consumption, smoking, and sleep duration).

**Results:**

The most robust associations were found between subjective caregiver burden and health behaviours, whereby caregivers reporting a higher burden reported less physical activity (Incidence Rate Ratio [IRR]:0.41; 95% CI 0.35-0.49), more alcohol consumption (IRR: 1.46; 1.20-1.77), greater smoking intensity (IRR: 1.29; 0.95-1.73), and a higher likelihood of insufficient sleep duration (Odds Ratio [OR]: 4.98; 1.58-15.74). Caregivers, who reported high objective burden, i.e. invested long hours in caregiving, were more prone to partake in health adverse behaviours, in particular greater alcohol consumption. Results also suggested that caregivers who were satisfied in their role and who received social support in caregiving were more likely to be physically active.

**Conclusion:**

Caregivers suffering from high emotional and time burden may benefit from both practical and psychological support. This support may release resources enabling individuals to partake in health promoting behaviours, or to develop coping strategies to better deal with the burden of caregiving.

## Background

The robust evidence for the detrimental effect of caregiver burden on health is worrisome (Beach, Schulz, Yee, & Jackson, 2000; Fekete, Tough, Siegrist, & Brinkhof, 2017; Schulz & Sherwood, 2008), particularly in the face of a rising global demand for care, and the society’s growing dependence on the ‘invisible healthcare system’ of informal caregivers. Informal care describes the non-professional and unpaid care for persons with long-term care needs by family members, friends, neighbours or other persons. Long-term caregiving may present itself as burdensome and therefore damaging for health for many caregivers. In contrast, the positive psychological returns of caregiving, such as satisfaction, fulfilment and purpose in life, have been associated with beneficial effects on health (Beach et al., 2000). It is therefore questionable whether it is the act of caregiving *per se,* or rather the caregiver’s appraisal of the individual situation, which is detrimental for health (Martire & Schulz, 2001). For example, a study from Switzerland found that the perceived burden of caregiving had more detrimental effects on caregivers’ health than did the time demands associated with caregiving duties (Fekete et al., 2017). This is in line with Lazarus and Folkman's transactional model of stress and coping, which emphasizes that the appraisal of a stressful event is more important that the event itself (Folkman, 2011). Fuelling the debate are several studies which found a decreased mortality risk for caregivers as compared to non-caregivers although selection bias, whereby healthy individuals are more likely to take on the caregiving role, cannot be excluded (Brown et al., 2009; Kaschowitz & Brandt, 2017; O’Reilly, Rosato, Maguire, & Wright, 2015). It is instrumental to health care policy to unravel the underlying pathways between caregiving and caregiver health to provide targets of intervention for optimizing the health of informal caregivers.

Health behaviour is one possible intermediate factor on the hypothesized pathway between the caregiver experience and health, and research suggests that not only the caregivers’ health, but also that the ability of the caregiver to provide long term care is improved if the caregiver is enacting health promoting behaviours (Kaschowitz & Brandt, 2017). It is assumed that a positive caregiver experience leads to health promoting behaviours, whereas a negative experience increases the risk of adverse behaviours. Health adverse behaviour in the context of this study include smoking, excessive alcohol consumption and less than recommended sleep duration. In contrast, health promoting behaviour, which is any behaviour which delivers health benefits and otherwise protects individuals from illness, include physical activity and fruit and vegetable consumption. The negative aspects of the caregiver experience can be described by the subjective and the objective burden. The subjective burden refers to the psychological or emotional strain resulting from caregiving responsibilities (Zarit, Reever, & Bach-Peterson, 1980), whereas the objective burden refers to the time burden, the number of activities assisted by the caregiver, or the amount of support received in caregiving. Importantly, informal caregiving may also be perceived as satisfying and fulfilling, thus resulting in a positive caregiver experience. These experiences of caregiving are not mutually exclusive and individual caregivers may experience both positive and negative emotions simultaneously, independent to the time in which they invest in caregiving duties. However, current literature and caregiver theory both suggest that those at the highest risk of subjective burden are those with the highest objective burden (Pearlin & Schooler, 1978), (Kim, Chang, Rose, & Kim, 2012). Different dimensions of health behaviour, such as diet, physical activity, smoking and alcohol consumption, may be affected in response to the limitations and strain, or positive aspects experienced by caregivers in everyday life. Both, the subjective and the objective burden may present distinct challenges for the enactment of health promoting behaviours, while the positive aspects of caregiving might facilitate and support the implementation of healthy behaviours. For example, time constraints due to long hours of caregiving may inhibit a caregiver's ability to take part in physical activity, lead to poorer nutrition, and the neglect of their own health care appointments (Burton, Newsom, Schulz, Hirsch, & German, 1997; Burton, Zdaniuk, Schulz, Jackson, & Hirsch, 2003; Mochari-Greenberger & Mosca, 2012). Whereas the stress related to subjective burden may promote risky health behaviours, such as excess alcohol consumption or chain smoking, as a way of coping with the challenging situation (Park & Iacocca, 2014). Positive affect, which can be provoked when appraising a situation to be satisfying or fulfilling, has been linked to health promoting behaviours (Fredrickson, Arizmendi, & Van Cappellen, 2020). It is therefore expected that caregivers who perceive their situation as more rewarding, are likely to have more of the psychological resources needed in order to follow a healthy lifestyle. Despite the wealth of evidence highlighting the negative relationship between chronic stress and adverse health behaviour, and positive emotions and health promoting behaviour, there is currently little evidence for the association between the caregiver experience and health behaviours, and available evidence is mainly concerned with ageing populations (Burton et al., 2003; Sisk, 2000).

In light of these research gaps, this study aims to investigate the association of objective and subjective caregiver burden, and the positive aspects of caregiving with health behaviours (physical activity, smoking, alcohol consumption, fruit and vegetable consumption, sleep duration) in middle-aged informal caregivers. The study is set in the longitudinal pro-WELL study that included informal caregivers and their partners with a physical disability, namely spinal cord injury (Fekete et al., 2017). A spinal cord injury is caused by a traumatic or non-traumatic event and leads to complete or partial loss of movement and sensation below the lesion level resulting in impairments in health and functioning. Informal caregiving is common in the context of spinal cord injury with up to 60% of persons indicating dependence on assistance from informal caregivers (Kemp, 2002). We hypothesize that higher levels of objective and subjective caregiver burden are associated with adverse health behaviours and that more positive caregiver experiences are associated with health promoting behaviours.

## Methods

### Sampling frame and participants

This study is based on data from the pro-WELL study (Fekete et al., 2017), which is a nested study within the community survey of the Swiss Spinal Cord Injury Cohort Study (SwiSCI). This sampling frame included a representative population of 1922 persons aged over 16 years with traumatic or non-traumatic spinal cord injury living in Switzerland (Brinkhof et al., 2016; Fekete et al., 2015). Of the 1922 SwiSCI participants, 676 persons were eligible for the pro-WELL study and 133 persons with spinal cord injury and their partners participated in the baseline assessment (response rate 19.7%). Details on inclusion criteria, recruitment outcomes, participation rates, and non-response are reported in the pro-WELL cohort profile (Fekete et al., 2017).

### Study design

Pro-WELL is a longitudinal community survey with three measurement waves (baseline; month 6; month 12) with the main objective to investigate the psychosocial determinants of wellbeing in persons with spinal cord injury and their caregiving partners. Given that the present study is mainly focused on the caregiving partners and that the main outcome (health behaviour) has only been measured in the baseline assessment, this analysis utilized cross-sectional baseline data from caregiving partners of persons with spinal cord injury (n=133). The baseline assessment was carried out between May 2015 and January 2016 and data were collected by means of standardized telephone interviews, paper-pencil or online questionnaires (Fekete et al., 2017), potential bias resulting from different data collection methods was tested, and there was no significant differences found between the different groups.

### Ethics statement

The study protocol and all measurements were approved by the Ethical Committee of Northwest and Central Switzerland (document EKNZ 2014-285). Regulations concerning informed consent and data protection were strictly observed and all participants signed an informed consent form.

### Measures

#### Health behaviours

Physical activity was assessed by information on weekly frequency (never or almost never, 1–2 times a week, 3–4 times a week, 5 times a week or more) and duration of physical activity (less than 30 min, between 30 and 60 min, between 1 and 2 h, over 2 h) (Andrich et al. 2012). These items were combined to derive the average duration of physical activity per day (in minutes). Alcohol consumption was measured using information on frequency of drinking occasions and amount of drinks consumed per drinking occasion (Gmel et al., 2012), which enabled us to compute average daily grams of alcohol consumed (BAG BfG, 2016). Smoking status was evaluated by gathering information on current smoking status (current smoker, ex-smoker, never smoker) taken from the International Spinal Cord Injury pulmonary Function Basic Data Set (Biering-Sørensen et al., 2012). Smoking intensity was assessed by information on mean daily amount of consumed tobacco products (cigarettes, cigars/cigarillos/cheroots; pipes), weighted by type of product (e.g. cigarettes: factor 1, cigars et al.: factor 3, pipes: factor 3.5) in order to calculate the mean daily consumption (Royal College of General Practitioners, 2014). Fruit and vegetable consumption was evaluated using items taken from the Swiss Health Survey by asking participants about the number of portions per day (1 or less, 2, 3, 4, 5 or more) and the amount of days with fruit or vegetable intake per week (never or almost never, 1–2 days per week, 3–4 days per week, 5–6 days per week, daily) (Statistics FOo, 2013; Winkler and Döring, 1998). Information was combined in order to estimate the amount of portions of fruit or vegetable consumed per week (Fekete et al., 2015). Sleep duration was assessed with an item asking about the average hours of sleep per night. Information was dichotomized into seven or more hours of sleep per night vs. less than seven hours sleep per night, based on sleep foundation recommendations for optimal sleep duration (Hirshkowitz et al., 2015).

#### Caregiver experience

*Objective caregiver burden* was assessed with information on amount of support provided for activities of daily living and instrumental activities of daily living, daily hours of caregiving and perceived social support in caregiving. Amount of activities of daily living support was assessed using six items from the Personal activities of daily living Scale provided information on support provided by caregivers with getting in and out of bed; using the toilet; dressing; bathing or showering; eating; and mobility within the home. An additional five items captured information on help with Instrumental activities of daily living, including help with doing the shopping; doing housework; managing money and paying bills; preparing meals; and providing transportation. Response options for the eleven items on type of help provided were ‘no help provided’ (0), ‘some help provided’ (1) and ‘much help provided’ (2). A sum score ranging from 0–12 for ADL and 0–10 for Instrumental activities of daily living was created (Schofield et al., 1997). Daily hours of caregiving were assessed as the number of hours invested per day in caregiving tasks. For analysis purposes, this variable was dichotomized as to discriminate those with a low burden (< 3 h/day) from those with high burden (≥3 h/day) (Hirst, 2003). In order to assess perceived social support in caregiving, a further item on whether caregivers received support in caregiving (yes/no) was evaluated.

*Subjective caregiver burden* was assessed using the Zarit Burden Interview (ZBI) short form, which captures personal feelings of strain resulting from the caregiving role (Zarit et al., 1980; Bédard et al., 2001). For example, participants were asked whether they experienced feelings of anger or strain, and whether the caregiving role had impinged on other areas of their lives. The five-point response scale includes the options never; rarely; sometimes; frequently; or nearly always. A sum score ranging from 0–48 was calculated. This variable was dichotomized in multivariable analysis based on Schreiner et al. (Schreiner et al., 2006) in order to identify those with high subjective burden, defined as sum scores >12. In our sample, this cut-off distinguishes the 20% of caregivers with the highest ZBI score from those with a lower ZBI score in investigating the association of caregiver burden with health behaviour. We further included one item on whether there was a wish for more support (yes/no).

*The positive aspects of caregiving* were assessed with one item on whether the caregiver experience provided a feeling of satisfaction. Response options ‘strongly disagree’, ‘disagree’, ‘agree’ and ‘strongly agree’ were dichotomized into agree vs. disagree.

#### Confounders

The identification of potential confounders was informed by current evidence and by directed acyclic graphs (DAGs; www.dagitty.net). Utilizing DAGs enables the identification of ‘true’ confounders which can subsequently be tested and validated in bivariate analysis. Age, gender, financial hardship, employment status (having paid work vs. not having paid work) and occurrence of a stressful life event in the last 6 months were identified as relevant confounders and were therefore included into multivariate models. Financial hardship was assessed with an item asking participants how they evaluate the availability of financial resources on a 5-point scale ranging from ‘very scarce’ to ‘lasts very well’.

### Statistical analysis

Analyses were conducted using STATA version 16.1 for Windows (College Station, TX, USA). Distribution of health behaviour variables in both persons with spinal cord injury and in caregiving partners were evaluated and dyadic concordance in health behaviours was assessed using multi-level models to compute within- and between-dyad variation. Intra-class correlations (ICCs) were evaluated to investigate how similar different variables were within dyads, with values closer to 1 indicating higher correlation within the dyad. Multivariate regression was also used to examine the difference in health behaviours between caregivers and persons with SCI, models were adjusted for age and sex. Descriptive analysis was performed with crude data, excluding all cases with missing values.

Regressions were applied in order to derive unadjusted and adjusted estimates of associations between the caregiver experience and health behaviours. For positive, right-skewed, continuous dependant variables with frequent values of zeros (i.e. daily average duration of physical activity, daily grams of alcohol, smoking intensity) zero-inflated Poisson models (ZIP) were fitted. For binary outcomes (i.e. smoking status and sleep duration), ordinary logistic regressions were applied, and for the continuous score of average daily portions of fruit and vegetables, linear regression was applied. Two subsequent models were computed: Model 1 was unadjusted; Model 2 was adjusted for age, gender, financial hardship, employment status and occurrence of a stressful life event in the last 6 months. *P values* were computed using equal fraction-missing-information (FMI) tests (Li et al., 1991). To account for item non-response in predictor and control variables, the multivariable analysis used multiply imputed data that were derived by multiple imputation using chained equations (MICE) (White et al., 2011). Outcome variables were not imputed. Selection bias due to unit non-response has been shown to be negligible and therefore not accounted for in data analysis (Fekete et al., 2017).

## Results

Basic characteristics of the pro-WELL sample are displayed in Table 1. The large majority of caregiving partners were female (74%) with a mean age of 50.2 years. About 70% of partners were involved in paid employment on top of their caregiving duties, while 60% of care-receivers were in paid work. Mean net equivalence household income was between 4300 CHF to 4600 CHF per month. Caregivers and care-receivers had been in formal educations for on average 14 years. In general, caregivers provided 1.7 h a day of informal care, and reported an average subjective caregiver burden score of 7.4 (range 0-48). Almost half of caregivers were satisfied with their role as a caregiver and two thirds received social support in caregiving, although 16% indicated a need for more support. On average, caregivers had provided almost 18 years of care to their partners.

**Table 1.** Characteristics of the pro-WELL sample (*N *= 266).

	Caregiving partner (*N* = 133)		Persons with spinal cord injury (*N* = 133)		ICC (95% CI)
Characteristic[missing value]	*n* (%)	Mean (SD); Median (IQR)	*n* (%)	Mean (SD); Median (IQR)	Within-dyad comparison
Sociodemographic characteristics					
Age (in years) [0,0]		50.2 (10.1); 52.0 (16.0)		51.7 (9.4); 53.0 (16.0)	0.79 (0.72, 0.84)
Female [0,0]	98 (73.7)		35 (26.3)		-
Education (in years) [7,2]		14.0 (3.1); 13.5 (4.0)		13.9 (3.2); 13.0 (4.0)	0.27 (0.14, 0.46)
Net equivalence household income (CHF)[17,19]		4376.3 (1567.9); 4500.0 (2031.3)		4585.0 (1493.3); 4583.3 (2000.0)	0.62 (0.50, 0.73)
Financial hardship [5,]	45 (35.2)		44 (34.1)		0.59 (0.37, 0.78)
Paid employment [0,0]	94 (70.7)		79 (59.4)		0.27 (0.09, 0.57)
**Lesion characteristics**					
Lesion severity [2]					-
Incomplete paraplegia	N/A		45 (34.4)		
Complete paraplegia	N/A		49 (37.4)		
Incomplete tetraplegia	N/A		24 (18.3)		
Complete tetraplegia	N/A		13 (9.9)		
Aetiology [3]					
Traumatic	N/A		109 (83.8)		
Non-traumatic	N/A		21 (16.2)		
**Objective caregiver burden**					-
Duration of daily care (in hours) [8]		1.7 (3.3); 1.0 (2.0)		N/A	
Social support in caregiving [10]	82 (66.7)		N/A		
Duration of caregiving (in years) [7]		17.9 (10.9); 15.5 (17.0)		N/A	
Number of activities of daily living tasks where assistance is given (range 0-12) [11]		2.0 (2.8); 1.0 (3.0)		N/A	
Number of instrumental activities of daily living tasks where assistance is given (range 0-10) [10]		3.6 (2.8); 3.0 (4.0)		N/A	
**Subjective caregiver burden**					-
Zarit Burden Interview (range 0-48) [1]		6.6 (7.0); 4.0 (9.0)			
Wish for more support in caregiving (yes)[16]	19 (16.2)				
**Satisfaction with caregiving** [23]					-
Satisfied	53 (48.2)				
Dissatisfied	57 (51.8)				

Abbreviations*: CHF* Swiss Francs*; SD* Standard Deviation; *IQR* Interquartile Range; *CI* Confidence Interval.

Table 2 demonstrates health behaviour variables in persons with SCI and in their caregiving partners. We observed clear differences in health behaviours between persons with spinal cord injury and caregiving partners. In general, caregiving partners were more physically active, but ate less fruit and vegetables, and smoked more often, albeit with a lower intensity than persons with spinal cord injury. Furthermore, results point at slightly higher alcohol consumption in persons with spinal cord injury. Intra-class correlations revealed that smoking status was similar within the couple, all other health behaviours did not seem to be correlated within couples.

**Table 2.** Health behaviours in persons with SCI and their caregiving partners: prevalence, dyadic coherence and differences.

	Missings	Caregiving partner *N*=133	Persons with spinal cord injury *N*=133	Within-dyad comparison	Difference between partners and persons with SCI (adjusted for age and sex)	
Continuous measures		*Mean (SD); Median (IQR)*	*Mean (SD); Median (IQR)*	*ICC (95% CI)*	*Effect size (95% CI)*	*p value*
Physical activity (minutes/day)	[0,0]	22.3 (27.6); 19.3 (22.5)	16.8 (22.8); 9.6 (19.3)	0.12 (0.03, 0.40)	1.35 (1.27-1.44)^a^	<0.001***
Alcohol consumption (grams/day)	[9,5]	7.6 (10.5); 4.4 (9.2)	9.0 (10.6); 4.4 (9.9)	0.20 (0.08, 0.41)	1.06 (0.96-1.16)^a^	0.268
Smoking intensity (tobacco consumed/day)	[9,9]	3.0 (6.1); 0.0 (1.5)	3.2 (7.6); 0.0 (0.0)	0.36 (0.23, 0.53)	0.68 (0.57-0.80)^a^	<0.001***
Fruit and vegetable consumption (portions/week)	[13,16]	22.0 (12.0); 21.0 (14.0)	28.7 (11.8); 28.0 (16.5)	0.09 (0.01, 0.48)	−9.93 (−13.4-6.5)^b^	<0.001***
**Dichotomous measures**		*n (%)*	*n (%)*	*ICC (95% CI)*	*Odds ratio (95% CI)*	*p value*
Currently smoking	[8,8]	38 (30.4)	29 (23.2)	0.62 (0.38, 0.81)	1.95 (1.00-3.80)^c^	0.051
Short sleep duration (less than 7h/night)	[5,4]	31 (24.0)	41 (30.8)	0.20 (0.04, 0.59)	0.77 (0.44-1.40) ^c^	0.366

Abbreviations: CI: confidence interval; ICC: Intra-class correlation; SD: standard deviation; IQR: interquartile range.

^a^ Incidence rate ratio from zero-inflated Poisson regression.

^b^ Coefficient from linear regression.

^c^ Odds ratio from logistic regression.

*p* < 0.05*, *p*<0.01**, *p*<0.001***.

Table 3 shows results from unadjusted and adjusted analyses of the association between caregiver experience and health behaviours. In general, results were in the hypothesized direction, i.e. that less objective and subjective caregiver burden and higher caregiver satisfaction were associated with higher likelihood of reporting health promoting behaviours and lower likelihood of reporting health adverse behaviours.

**Table 3.** Associations between the caregiving experience and health behaviours: Results from multivariable analysis.

			Health promoting behaviours			Health adverse behaviours			
			Physical activity	Fruit and vegetable consumption		Alcohol consumption	Smoking intensity	Currently smoking	Low sleep duration
	Measurement unit		(Mins/day)	(Portions/week)		(Grams/day)	(Tobacco items/day)	(Yes/No)	(<7 hrs/≥7 hrs)
	Effect size		IRR (95% CI)^a^	Coefficient (95% CI)^c^		IRR (95% CI) ^a^	IRR (95% CI) ^a^	OR (95% CI)^b^	OR (95% CI) ^b^
**Objective caregiver burden**	**Amount of support in activities of daily living**								
	Model 1	0–12	0.98 (0.97-1.00)	−0.50 (−1.27-0.28)		1.07 (1.03-1.11)**	1.05 (1.01-1.08)*	1.08 (0.94-1.23)	1.16 (1.01-1.33)*
	Model 2	0–12	0.96 (0.94-0.98)***	−0.70 (−1.51-0.10)		1.04 (1.00-1.09)	1.05 (1.00-1.09)*	1.09 (0.94-1.28)	1.14 (0.98-1.32)
	**Amount of support in instrumental activities of daily living**								
	Model 1	0–10	0.98 (0.96-1.00)*	−0.59 (−1.39-0.21)		1.05 (1.02-1.08)***	1.01 (0.98-1.05)	1.06 (0.93-1.22)	1.14 (0.99-1.33)
	Model 2	0–10	0.97 (0.95-0.99)***	−0.49 (−1.33-0.35)		1.02 (0.99-1.05)	1.03 (0.99-1.06)	1.06 (0.91-1.23)	1.11 (0.94-1.31)
	**Hours of caregiving**								
	<3 h of daily care		Reference	Reference		Reference	Reference	Reference	Reference
	Model 1	>3 h of daily care	1.13 (1.04-1.24)**	−5.28 (−11.30-0.73)		1.76 (1.47-2.11)***	1.15 (0.92-1.44)	1.26 (0.48-3.30)	2.13 (0.79-5.74)
	Model 2	>3 h of daily care	1.10 (1.00-1.21)	−4.88 (−10.85-1.08)		1.46 (1.21-1.77)***	1.13 (0.85-1.50)	1.41 (0.49-4.10)	2.02 (0.67-6.10)
	**Social support in caregiving**								
	Social support		Reference	Reference		Reference	Reference	Reference	Reference
	Model 1	No social support	0.77 (0.70-0.85)***	−0.98 (−5.71-3.76)		0.85 (0.68-1.06)	1.04 (0.85-1.28)**	0.96 (0.42-2.20)	1.42 (0.61-3.30)
	Model 2	No social support	0.78 (0.70-0.87)***	−1.97 (−6.51-2.58)		0.80 (0.64-1.00)	0.97 (0.74-1.28)**	1.12 (0.47-2.66)	1.77 (0.71-4.38)
**Subjective caregiver burden**	**Low subjective burden (ZBI-S ≤12)**		Reference	Reference		Reference	Reference	Reference	Reference
	Model 1	High burden (ZBI>12)	0.45 (0.38-0.52)***	−1.09 (−5.98-3.80)		1.67 (1.40-2.00)***	1.41 (1.14-1.73)**	1.94 (0.74-5.09)	3.77 (1.41-10.04)**
	Model 2	High burden (ZBI>12)	0.41 (0.35-0.49)***	−0.18 (−5.20-4.84)		1.46 (1.20-1.77)***	1.29 (0.95-1.73)	2.58 (0.86-7.72)	4.98 (1.58-15.74)**
	**Wish for more support (Yes/no)**								
	No wish for more support		Reference	Reference		Reference	Reference	Reference	Reference
	Model 1	Wish for more support	0.84 (0.74-0.95)**	−3.18 (−8.42-2.07)		1.03 (0.81-1.32)	1.05 (0.80-1.39)	1.10 (0.38-3.19)	2.39 (0.86-6.60)
	Model 2	Wish for more support	0.97 (0.85-1.11)	−2.47 (−7.85-2.91)		0.86 (0.67-1.09)	1.27 (0.94-1.73)	1.17 (0.36-3.81)	2.77 (0.85-9.01)
**Caregiver satisfaction**									
	Satisfied		Reference	Reference		Reference	Reference	Reference	Reference
	Model 1	Dissatisfied	0.70 (0.60-0.81)***	0.88 (−3.76-5.52)		1.00 (0.82-1.21)	0.72 (0.57-0.92)*	1.08 (0.49-2.37)	0.65 (0.28-1.50)
	Model 2	Dissatisfied	0.66 (0.57-0.77)***	0.25 (−4.63-5.13)		1.15 (0.91-1.46)	0.53 (0.38-0.73)***	1.41 (0.57-3.46)	0.85 (0.33-2.20)

Abbreviations*: ADL* activities of daily living, *CI* confidence interval, *IRR* incidence rate ratio, *OR* odds ratio, *ZBI-S* Zarit Burden Interview short form

^a^ Effect sizes from zero-inflated Poisson regression; ^b^Effect sizes from logistic regression; ^c^Effect sizes from linear regression.

*p* < 0.05*, *p*<0.01**, *p*<0.001*** *p* from Equal-Fraction-Missing information (FMI) test. Missing values were imputed by multiple imputation. Bootstrap 95% CI are displayed for Model 2.

Model 1 was unadjusted; Model 2 was adjusted for age, gender, financial hardship, employment status and occurrence of a stressful life event in the last 6 months.

Several of the indicators for objective burden showed consistent associations with health behaviours. Caregivers who provided more support in activities of daily living and instrumental activities of daily living showed on average lower physical activity (IRR 0.96; 0.94-0.98, IRR 0.96; 0.94-0.98 respectively) and higher smoking intensity than caregivers who provided less support. Those with social support in caregiving further reported higher levels of physical activity (IRR 1.28; 1.15-1.43). Individuals who invested over 3 h in caregiving per day reported a higher alcohol consumption than those who invested less time in care, although they were also more likely to participate in more physical activity (IRR 1.10; 1.00-1.21).

Caregivers with a higher subjective burden were less than half as likely to partake in physical activity (IRR 0.41; 0.35-0.49), consumed nearly 50% more alcohol per day (IRR 1.46; 1.21-1.77) and were almost five times more likely to sleep for less than seven hours per night (OR 4.98; 1.58-15.74) than those with a lower subjective burden.

Those caregivers who reported few positive aspects of caregiving, i.e. dissatisfaction with the caregiving role, reported lower levels of physical activity than those who were satisfied (IRR 0.66; 0.57-0.77) and higher likelihood of smoking (OR 1.41; 0.57-3.46), albeit with a lower intensity than those who were satisfied.

## Discussion

This study provides evidence for the connection between health behaviours and the caregiving experience. In general, our findings support the hypothesis that a higher objective and subjective burden, and a lower satisfaction with caregiving are associated with less health promoting and more health adverse behaviours. More specifically, caregiving partners with a high subjective burden reported higher levels of alcohol and tobacco consumption, lower levels of physical activity and shorter sleep duration. Moreover, the caregiver experience was generally associated with physical activity; showing either raised or reduced levels of physical activity for respectively positive and negative aspects of caregiving. These findings are novel for the caregiver health literature and may reveal an important pathway linking caregiver burden and lack of satisfaction with the caregiver role to health outcomes.

What is particularly novel to the literature is our finding that positive appraisals, such as satisfaction with the caregiver role are generally associated with healthy behaviour. Furthermore, we confirmed previous findings that caregiver burden is linked to more health adverse behaviour; this enforces the notion that a comprehensive assessment of the caregiving experience must include both, positive and negative experiences and go beyond the assumption that caregiving is a burden. For many individuals, caregiving is appraised as a positive, fulfilling and satisfying experience with subsequent health enhancing effects, as documented for example for mental health (Fekete et al., 2017). More specifically, results suggest that a positive appraisal of the caregiving role may lead to other positive outcomes, such as increased levels of fulfilment and purpose in life, that in turn provide resources for health promoting behaviours (McEwen and Lasley, 2002). Indeed, the response to stress, the appraisal of potential stressful situations, and the use of health behaviours to combat stress, is seen to be very individualized. For example, physical activity may be seen for some as another demand on their time and lead to higher psychological distress, whereas for others it presents a coping strategy to mitigate the negative effects of stress (Stults-Kolehmainen and Sinha, 2014). It is assumed that physical activity creates resources, which can be used to appease stressful situations as several studies have found that more active individuals often have higher levels of activity in times of increased stress (Krueger and Chang, 2008). In our study we see the most robust associations between caregiving and physical activity, but interestingly, there is not an absolute trend towards less physical activity for caregivers with a higher burden. In fact, data reveal that those who invest a lot of time in caregiving are more physically active than those with a lower time demand are. In this sense, it could be that health promoting behaviours, such as physical activity, buffer the potentially stressful effect of highly intensive caregiving situations on perceived burden, enabling these caregivers to appraise their situation as less stressful. Moreover, it may also be the case that caregivers’ rate carrying out caregiving activities as physical activity. Although research in the field of caregiving and health behaviours is scarce, our results are in line with current evidence on the impact of chronic stress, like that associated with prolonged caregiving, on health adverse behaviours (Beach et al., 2000; Krueger and Chang, 2008; Fuller-Jonap and Haley, 1995; Heikkilä et al., 2013). Perceived stress is associated with less physical activity and more sedentary behaviour (Stults-Kolehmainen and Sinha, 2014; Vitaliano et al., 2002; Marquez et al., 2012), increased smoking (Ng and Jeffery, 2003), poorer nutrition (Vitaliano et al., 2002; Ng and Jeffery, 2003), and higher alcohol consumption. The addition of the caregiving experience as additional productive activity (Heikkilä et al., 2013; Fransson et al., 2012) further demonstrates that juggling multiple roles, especially if those roles are appraised as emotionally stressful, may be detrimental to health behaviour. The link between stress, coping and health behaviour is thought to be due to the damaging effect of stress on an individual’s level of self-efficacy and self-control, making the temptation to indulge in unhealthy behaviours too hard to resist, and encouraging individuals to partake in health adverse behaviours as a form of mood self-management and coping response (Pearlin and Schooler, 1978).

## Practical implications

Our findings suggest that caregivers bearing a high subjective burden present an important target population for interventions aimed at altering health behaviours. However, there are special considerations, which need to be made in the design of tailored programmes. For example, caregivers may need different prescriptions for physical activity than other populations, regimes that concentrate on shorter bouts of physical activity rather than long training sessions have been seen to be more successful in caregiving population (Marquez et al., 2012), potentially because of difficulties to arrange longer time slots between diverse responsibilities. Also by reducing health adverse behaviours in order to improve general health, caregivers may lose an emotion-focused coping method, which would need to be replaced through education and training, or through the adoption of a health promoting behaviour as a coping tool (Pearlin and Schooler, 1978; Krueger and Chang, 2008; Revell et al., 1985; Warburton, 1992). Health behaviours could be indirectly targeted by psychoeducational or psychotherapeutic interventions aimed at reducing the subjective caregiver burden and increasing levels of social support. Alternatively, by practical measures which relieve caregivers of performing certain caregiving tasks (Sörensen et al., 2002). Training should be offered to all caregivers at the transition into caregiving, but also throughout the caregiving life course as caregivers will most likely need to adapt to the dynamic nature of the care-receivers condition, and to the process of ageing. This training should highlight the importance of self-care and also provide both practical and emotional support in dealing with the demands of informal caregiving. One important aspect of interaction between the healthcare and social system, and the caregiver could be a regular and structured needs assessment to identify situations, which are especially burdensome or distressing for the caregiver and may lead to adverse health behaviours. Health promoting behaviours may additionally enable individuals to meet the challenges faced as caregivers prolonging the time that they are able to provide the necessary support to their partners, reducing the need to institutionalize care-receivers, and resulting in longer term and better quality care. In the case of spinal cord injury, physical strength is for example often needed in order to support care-receivers in transfers in and out of the wheelchair, and better physical condition has been associated with improvements in the technique of the transfer, leading to less risk of injury for both, the caregiver and the care -receiver (Kinugasa et al., 1995; Bardak et al., 2012). This means that if the negative effect of caregiver burden on health behaviours was alleviated in this population, there would be benefits for both the caregiver and the care-receiver. Given that there also seems to be evidence for dyadic concordance in a selection of the health behaviours under study, we could also infer that couple-based behaviour change programmes would be beneficial in this population (Arden-Close and McGrath, 2017).

## Strengths and limitations

A major strength of this study is the use of a wide-ranging set of variables to describe the caregiver experience, which capture not only the subjective and objective nature of caregiver burden but also positive aspects. In addition, we were able to investigate a large array of different health behaviours, capturing health promoting and health adverse behaviours. All associations were tested using multivariate models, which took relevant confounders into account. Furthermore, the pro-WELL study was nested in a large cohort study, showing good representation of the source population of care-receivers in terms of socio-demographic and lesion characteristics (Fekete et al., 2017). Yet, a possible limitation includes volunteer bias with respect to caregiver burden or associated health status, as the couples least burdened by the caregiver situation may have been more likely to participate than couples with high caregiver burden. Moreover, the self-report of caregiver experience and health behaviours may have been subject to recall bias and social desirability. Furthermore several of the health behaviour measures have not been validated which may lead to inaccuracy of measurement, however most have been used in large countrywide studies in Switzerland and Germany. Misclassification of health behaviour is also conceivable, although this bias is thought to be largely independent of caregiving variables, and thus result in attenuation rather than inflation of effects sizes. Additionally, we have made certain assumptions about the reasons why persons might undertake health promoting or adverse behaviours, in future research it would be beneficial to directly measure whether individuals partake in certain behaviours as a response to stress and as a coping mechanism. Furthermore, reviews on stress and health behaviour have suggested that there are complex causal pathways between the two concepts, which are unlikely to be untangled using cross-sectional data. Future studies using life course data on the caregiver experience may facilitate the evaluation of trajectories in health behaviour, also to address to whether a healthy lifestyle as such, affects appraisal of the caregiving situation. It may for example be that persons who adhere to a healthy lifestyle are able to appraise their caregiving situation as less stressful. Finally, these results may not be generalizable to other caregiving settings, as factors causing distress to caregivers are at least partly context specific. For example, hostile or aggressive behaviour by the care-receiver may be an additional exposure and stressor for health behaviour in caregivers for persons with cognitive impairments, such as Alzheimer disease.

## Conclusion

This study provides an important contribution to the literature on informal caregiving and health behaviour as it goes further than current evidence by including both positive and negative aspects of the caregiver experience as potential predictors of health behaviour. The reduction of the burden of care or the increase of positive aspects of caregiving is considered as a valuable strategy to indirectly support the promotion of favourable health behaviours in caregivers. Therefore, health policy may develop programmes involving both practical and emotional support, e.g. by caregiving relief through respite care or through psychological therapies. A structured needs assessment could assess the ability of the caregiver to provide care and identify situations, which are especially burdensome or damaging for healthy behaviours.

## Data Availability

The datasets analysed during the current study are available from the corresponding author on reasonable request.

## Abbreviations

Abbreviations: CHF: Swiss Francs; DAG: Directed acyclic graphs; FMI: Equal fraction-missing-information; ICC: Intra-class correlation; IRR: Incidence Rate Ratio; MICE: Multiple imputation using chained equations; OR: Odds ratio; SwiSCI: Swiss Spinal Cord Injury Cohort Study; ZBI: Zarit Burden Index; ZIP: Zero-inflated Poisson
